# Symptomatic Achilles Tendons are Thicker than Asymptomatic Tendons on Ultrasound Examination in Recreational Long-Distance Runners

**DOI:** 10.3390/sports7120245

**Published:** 2019-12-05

**Authors:** Bo Tillander, Håkan Gauffin, Johan Lyth, Anders Knutsson, Toomas Timpka

**Affiliations:** 1 Athletics Research Center, Linköping University, SE-581 83 Linköping, Sweden; hakan.gauffin@regionostergotland.se (H.G.); toomas.timpka@liu.se (T.T.); 2 Department of Orthopaedics, Linköping University, SE-581 83 Linköping, Sweden; 3 Research and Development Unit in Region Östergötland, SE-581 85 Linköping, Sweden; johan.lyth@regionostergotland.se; 4 Department of Medical and Health Sciences, Linköping University, SE-581 83 Linköping, Sweden; 5 Department of Radiology, Linköping University, SE-581 83 Linköping, Sweden; anders.knutsson@regionostergotland.se

**Keywords:** running, ultrasound, achilles tendon, middle-aged

## Abstract

There is a need for clinical indicators that can be used to guide the treatment of Achilles tendon complaints in recreational runners. Diagnostic ultrasound has recently been introduced for clinical decision support in tendon pain management. The aim of this study was to determine whether tendon thickness and morphological changes in the Achilles tendon detected in ultrasound examinations are associated with local symptoms in middle-age recreational long-distance runners. Forty-two Achilles tendons (21 middle-aged runners) were investigated by ultrasound examination measuring tendon thickness and a morphology score indicating tendinosis. The Generalized Estimating Equations method was applied in multiple models of factors associated with reporting a symptomatic tendon. Eleven symptomatic and 31 asymptomatic Achilles tendons were recorded. In the multiple model that used tendon thickness measured 30 mm proximal to the distal insertion, an association was found between thickness and reporting a symptomatic tendon (*p* < 0.001; OR 12.9; 95% CI 3.1 to 53.2). A qualitative morphology score was not found to be significantly associated with reporting a symptomatic tendon (*p* = 0.10). We conclude that symptomatic Achilles tendons were thicker than asymptomatic tendons on ultrasound examination among recreational long-distance runners and that the importance of parallel morphological findings need to be further investigated in prospective studies.

## 1. Introduction

Running is one of the most popular forms of exercise worldwide [1]. A common location of pain that restricts the ability to continue the exercise among recreational runners is the Achilles tendon [2,3]. A recent systematic review confirmed a high incidence (9–11%) and prevalence (6–10%) of Achilles tendinopathy (pain and dysfunction) in runners [4]. Pang and Ying 2006 [5] reported a mean Achilles tendon thickness of 5.1 ± 0.6 mm among asymptomatic subjects of different ages and no significant difference between dominant and non-dominant ankles, while there is evidence that external load influences tendon structural adaptive properties [6,7,8]. Jhingan et al. [9] found greater mid-tendon thickness in tendons that produced symptoms the following year among soccer players. The thickness of the Achilles tendon has also been found to be correlated with rupture risk and tendon abnormalities [10,11,12]. Moreover, ingrowth of new blood vessels into tendinopathic areas are often seen on imaging examinations and the ingrowth of accompanying nerves has been shown to be weakly related to pain symptomatology [13]. Hirschmuller et al. [14] reported that observation of such intratendinous microvessels in the Achilles tendons of healthy runners were prognostic for symptom development within one year. However, although Achilles tendon pain is a common reason for quitting running among middle-aged recreational long-distance runners at this performance level, little data are available that can be used to guide clinical interventions among runners in this age-group. Ultrasound techniques have recently been introduced for clinical decision support in the management of tendon complaints [15]. The fact that diagnostic ultrasound is non-invasive, inexpensive, and the Achilles tendon location is easy to visualize has, combined with the growing research evidence, made the examination popular among health care providers [16,17].

However, several studies have also demonstrated a high incidence of hypoechoic areas in the Achilles tendons of healthy runners without clinical signs and symptoms of tendinopathy [18,19,20,21]. Tendon abnormalities on imaging (tendinosis) do therefore not confirm that pain and dysfunction (tendinopathy) are generated by the tendon, which implies a risk for overtreatment. Furthermore, a normal tendon ultrasound does not rule out the tendon as the source of pain and dysfunction [22].

The aim of the study was to determine whether tendon thickness and qualitative morphological changes in the Achilles tendon detected in ultrasound examinations are associated with local symptoms in middle-aged recreational long-distance runners.

## 2. Materials and Methods

The study used a cross-sectional design. The design was approved by the research ethics committee in Linköping (Dnr 2015/163-31).

### 2.1. Participants and Recruitment

The study sample size estimation was based on the study reported by Ooi et al. 2015 [23]. Runners in this study displayed a mean sagittal tendon thickness of 5.2 mm (SD 0.7 mm) and the controls 4.6 mm (SD 0.7 mm). Thirty-eight percent of the tendons in this study were symptomatic providing a ratio of approximately two asymptomatic tendons per symptomatic tendon. Using these numbers, it was found that a study sample of 20 runners (40 tendons) was required to detect a difference in tendon thickness between symptomatic and asymptomatic tendons (power = 0.80, sign = 0.05, one-tailed independent samples *t*-test).

We recruited middle-aged (35–60 years of age) recreational runners from Lidingöloppet 30 k cross-country race (Stockholm, Sweden) in September 2017. Written information about the ultrasound examination was sent to those runners having volunteered to participate in a clinical study with residence closest to Linköping University Hospital. 21 runners agreed to participate. The runners’ characteristics are presented in Table 1.

### 2.2. Questionnaire

Before the ultrasound examination the participants signed a written consent and then answered the eight questions in the validated score for complaints referring to the Achilles tendon, VISA-A-S [24]. All questionnaires were filled in just before the ultrasound examination. The respondent described whether he or she had symptoms or not and which side(s) was affected. A VISA-score <90 was considered to be symptomatic. A score ≥90 has previously been reported to represent full recovery following Achilles tendinopathy [25,26]. 

### 2.3. Ultrasound Examination

**Figure 1:**
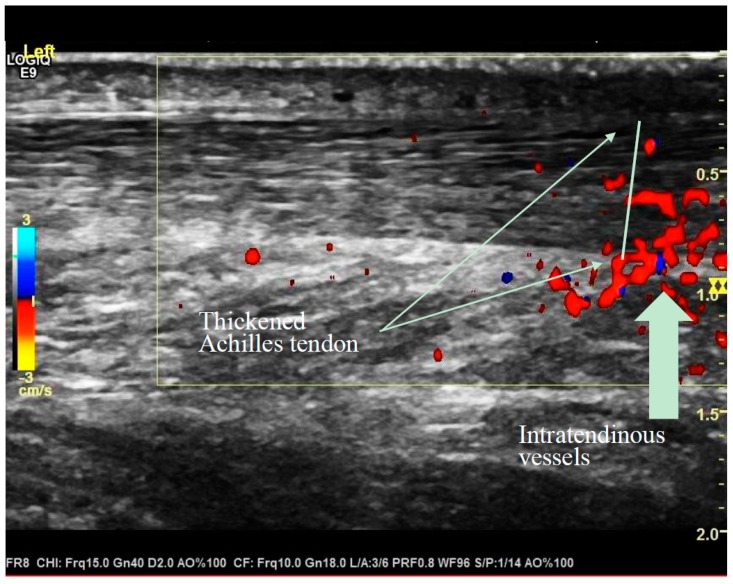
Thickened Achilles tendon and intratendinous vessels recorded at diagnostic ultrasound examination.

The Achilles tendons (*n* = 42) were examined four months after Lidingöloppet at the University Hospital of Linköping in January and February 2018. The ultrasound examinations were performed by one of the authors (AK) who is an experienced senior ultrasound specialist. At the examination, the runner lay on a bench in a prone position with both feet hanging outside the bench. As a routine, a gel is applied on the skin over the area which is examined with a probe. The examinations were recorded, stored and coded by numbers. Two months after the ultrasound examination, the stored coded data were assessed by AK (Figure 1).

### 2.4. Ultrasound Criteria

The recordings were evaluated as follows [27,28]:

*Primary clinical indicator:*

The sagittal thickness of the tendon 30 mm proximal to the insertion at the calcaneus bone.

*Secondary clinical indicators:*

1. Maximal thickness in the sagittal plane (mm).
2. Structure of the tendon and semi-quantitative evaluation of capillaries according to the modified Öhberg Score [29]).
3. Are there signs of tendinosis (dichotomous): yes or no?
4. Are there signs of bursitis (dichotomous): yes or no?

The criteria for the qualitative evaluations were according to the modified Öhberg Score [29] set as follows:

*Tendon structure and edema:*

0—normal structure (homogenous echogenicity);

1—light structural changes (discrete hypo-echogenic areas);

2—moderate structural changes (some well-defined hypo-echogenic areas);

3—severe structural changes (extended hypo-echogenic areas).

*Neovascularisation* (visible high blood flow): 

0—no neovascularization;

1—mild neovascularisation (a few solitary, blood vessels);

2—moderate neovascularisation (moderate quantity, mostly transversal blood vessels).

### 2.5. Tendinosis and Bursitis

Dichotomous evaluations (0–1) of the presence of tendinosis and bursitis signs were made by the experienced senior ultrasound specialist (A.K.). The tendinosis judgement included factors such as tendon structure, edema, neovascularization, and tendon thickness, while the bursitis judgement comprised signs of swelling in the retro-calcaneal bursa.

### 2.6. Qualitative Morphology Score

A combined morphology score ranging from 0 to 8 was constructed by taking the sum of tendon structure and edema (0–3), neovascularization (0–3), tendinosis (0–1) and bursitis (0–1).

### 2.7. Statistical Analysis

Pearson’s correlation coefficients (*r*) were calculated between the diagnostic indicators (maximal thickness, thickness of the tendon 30 mm proximal to the insertion at the calcaneus bone, relation between the 30 mm thickness and maximal thickness, and morphology score) and the potential confounding factors (age, weight, BMI, running mileage per week, and running experience).

Measures of ultrasound pathology/morphology were compared between asymptomatic and symptomatic Achilles tendons. Because of potential correlation between observations caused by two measures per person, the Generalized Estimating Equations (GEE) method was used to obtain *p*-values [30]. A normal distribution was used for continuous response variables and a binary distribution was used for categorical response variables. Categorical data were dichotomized into yes/no. In all GEE analyses side was used as a within-subject variable and the analyses were based on the robust estimator method using an unstructured working correlation matrix. *p*-values below 0.05 were considered statistically significant. 

The GEE method with a binary distribution with the same parameters as above was also used in two multiple models of factors associated with reporting a symptomatic tendon. The first multiple model used the maximal thickness of the Achilles tendon and morphology score as explanatory variables and the second model included thickness of the tendon 30 mm proximal to the insertion at the calcaneus bone and morphology score as corresponding variables. Having a symptomatic tendon (y/n) was used as the response variable in both multiple models. Associations were presented as odds ratio (OR) with 95% confidence intervals (CI). To support the interpretation of the results, post hoc analyses of differences in tendon thickness with regard to the presence of symptoms were performed separately for symptomatic tendons with and without morphological changes.

All statistical analyses were undertaken using SPSS version 25.0 (IBM Corp., Armonk, NY, USA).

## 3. Results

Eleven (26%) symptomatic (VISA-A-S score <90) and 31 (74%) asymptomatic Achilles tendons were recorded (3/21 runners with one symptomatic tendon and 4/21 runners with two symptomatic tendons). No statistically significant correlations between the clinical indicators (tendon thickness and morphology score) and the potential confounding factors (age, weight, BMI, running mileage per week, and running experience) were observed (Table 2).

The simple model analyses showed that the symptomatic Achilles tendons were thicker than the asymptomatic tendons measured as the maximum thickness of the tendon (*p* = 0.006; Cohen’s *d* 1.1), as the thickness at 30 mm proximal to the distal insertion (*p* < 0.001; Cohen’s *d* 1.7), and as the proportional relation between these measures (*p* = 0.01; Cohen’s *d* 0.8) (Table 3). There were qualitative signs of vessel in-growth, edema and tendinosis in both symptomatic and asymptomatic tendons (Table 3), but no statistical significant differences were found in the simple model analyses between symptomatic and asymptomatic tendons. Nine of the 31 asymptomatic tendons showed signs of tendinosis at the ultrasound examination.

The multiple model that included maximal tendon thickness as explanatory variable showed that neither the maximal thickness (*p* = 0.12) nor the morphology score (*p* = 0.80) were associated with reporting a symptomatic tendon. In the multiple model that instead used the tendon thickness 30 mm proximal to the distal insertion, a significant association was found between the tendon thickness and reporting a symptomatic tendon (*p* < 0.001; OR 12.9; 95% CI 3,1 to 53,2). The qualitative morphology score was not found to be significantly associated with reporting a symptomatic tendon (*p* = 0.10). 

A post hoc analysis showed that symptomatic tendons with and without morphological changes were thicker than the asymptomatic tendons when measured as the maximum thickness (with morphological change *p* = 0.04; without change *p* = 0.03) and 30 mm proximal to the distal insertion (with change *p* = 0.001; without change *p* = 0.02) (Table 4). The proportional thickness was greater in the symptomatic tendons with morphological changes than the asymptomatic tendons (*p* = 0.01), while the symptomatic tendons without morphological changes did not differ from the asymptomatic tendons with regard to this thickness measure (*p* = 0.29).

## 4. Discussion

The aim of this study was to determine whether tendon thickness and qualitative morphological changes in the Achilles tendon detected in ultrasound examinations are associated with local symptoms in middle-age recreational long-distance runners. We found that symptomatic Achilles tendons were significantly thicker 30 mm proximal to the distal insertion at the calcaneal bone compared to asymptomatic tendons in middle-aged recreational long-distance runners. No statistically significant differences between symptomatic and asymptomatic tendons were observed with regard to morphological findings at ultrasound examination, such as neo-vascularization, irregular tendon structure or edema.

A critical question is how the observed difference in thickness between symptomatic and asymptomatic tendons should be interpreted from a clinical perspective, and more specifically, how tendon thickness measured at ultrasound examination can be used to guide treatment choices. There is considerable evidence demonstrating that external load is one of the primary etiological factors that influences the structural properties of tendons [6,7,8]. Load has been shown to be both anabolic and catabolic for tendons [31] and could affect the tendon in a positive or negative way. With optimized load, tendon adaptation may occur resulting in a stronger and more load-tolerant tendon. Docking et al. [32] showed that the UTC echo-pattern of the Achilles tendon improved over a 5-month pre-season training period in Australian football players, representing increased fibrillar alignment, but without any difference in tendon thickness. Hullfish et al. [33] concluded that the thickening of the tendons in competitive runners represented adaptive structural changes and also Shaikh et al. [21] found increased tendon thickness in asymptomatic competitive runners. Ooi et al. [23] found that marathon running induced a significant change in Achilles tendon stiffness and Doppler signals. The increase in tendon dimensions may be a mechanism by which the tendon maintains sufficient mean cross-sectional area aligned fibrillar structure to still tolerate load [22,28,34].

However, excessively loading a non-adapted tendon may result in a negative response where the tendon transitions into a reactive tendinopathy [32]. Cook and Purdam have suggested continuum model of tendon changes and pathology [8], and also other theoretical models have been proposed that suggest tendon alteration to accumulate within the tendon to a point where it no longer can remodel to compensate for the area of disorganization. Abate et al. [35] described the “Iceberg theory” of tendon pathology where relative overload induces micro-ruptures within the tendon that sets of a cascade of changes in the extracellular matrix. Here, adequate thickening of the Achilles tendon in response to age and tendon stress has been described to be most critical in the structurally susceptible middle section [11]. This susceptibility (lower adaptive capacity) could explain our finding of that the difference in thickness between symptomatic and asymptomatic Achilles tendons was most significant at 30 mm proximal to the distal insertion to the calcaneal bone, i.e., in the section of the tendon with least nutritional provision.

A further question is how the qualitative morphology findings in this study should be interpreted from a clinical perspective [36]. Several studies have demonstrated a high incidence of hypoechoic Achilles tendons even in individuals without clinical symptoms of tendinopathy [18,19,20,21]. This is also analogous to findings in other areas of musculoskeletal complaints. For instance, Girisch et al. [37] could in asymptomatic shoulders detect morphological changes by ultrasound of the shoulder in people between 40 and 70 years of age. Our results are in accordance with these results and call for careful interpretation of morphological ultrasound findings. The post hoc analyses showed that the symptomatic tendons with morphological changes had a higher proportional thickness at 30 mm proximal to the distal insertion compared to the asymptomatic tendons, while the symptomatic tendons without morphological changes did not differ significantly from the asymptomatic tendons in this regard. This finding suggest a progression of pathology, where tendons exposed to excessive loads initially adapt by increasing their thickness, thereafter generate symptoms, and finally display morphological changes. The morphological findings may thus represent indications of non-reversibility of the pathological process, rather than being correlated to pain. However, due to the cross-sectional design of this study, this interpretation of the results needs to be confirmed in prospective studies.

The study limitations comprise its cross-sectional design, which warrants reproduction of the findings in a prospective study before wider use in clinical practice. The size of the study sample also needs to be considered. It should be noted that the power calculation used to determine the sample size was fitted to detect difference in thickness between symptomatic and asymptomatic tendons. Differences between symptomatic and asymptomatic tendons with regard to morphological findings may thus have not been detected and still exist.

## 5. Conclusions

Analysis of ultrasound examination data from middle-aged recreational long-distance runners showed that symptomatic Achilles tendons were significantly thicker 30 mm proximal to the distal insertion than asymptomatic tendons. Morphological signs of vessel in-growth, edema, and tendinosis were observed in both symptomatic and asymptomatic tendons. These results provide verification of that ultrasound techniques can be used for classifying and managing Achilles tendon pain. The clinically significant breakpoint for the Achilles tendon thickness measured at 30 mm proximal to its distal insertion and the importance of parallel morphological findings need to be further investigated in prospective studies.

## Figures and Tables

**Table 1:** Characteristics of 21 individuals examined by ultrasound.

Runner Characteristic	
Sex, n (%)	
Men	15 (71.4)
Women	6 (28.6)
Age, mean (SD)	47.5 (6.3)
Age, median (range)	49.0 (35.0–59.0)
BMI, mean (SD)	23.3 (2.0)
BMI, median (range)	23.6 (19.8–26.3)
Race time in minutes, mean (SD)	198 (39)
Race time in minutes, median (range)	188 (151–287)
Km/week average training distance, mean (SD)	28.8 (13.5)
Km/week average training distance, median (range)	30.0 (7.0–43.0)
Long distance training pace in minutes/km, n (%)	
4:30–4:59	2 (10.0)
5:00–5:29	4 (20.0)
5:30–6:29	11 (55.0)
>6:30	3 (15.0)
Years of running, n (%)	
<3 years	0 (0)
3–5 years	3 (15.0)
6–10 years	7 (35.0)
>10 years	10 (50.0)

Missing values: BMI (*n* = 1), Race time (*n* = 4), KM/week (*n* = 1), Training pace (*n* = 1), Years of running (*n* = 1).

**Table 2:** Correlations (correlation coefficient *r*) between ultrasound findings and runners age, weight, running mileage, and running experience (all *p* > 0.05).

Ultrasound Findings	Age	Weight	BMI	Mileage/Week	Running Experience
**Max. thickness**	0.16	0.16	0.05	0.17	0.02
**30 mm thickness**	0.09	0.15	0.02	0.29	−0.01
**30 mm/max, proportion**	−0.05	0.03	−0.01	0.23	0.01
**Morphology score**	0.24	0.14	0.09	−0.03	0.11

**Table 3:** Simple model analyses of ultrasound pathology/morphology in 42 Achilles tendons.

Ultrasound Pathology/Morphology	Asymptomatic n = 31	Symptomatic n = 11	*p*-Value
Maximal thickness in the sagittal plane in mm, mean (SD)	5.7 (1.0)	6.7 (0.8)	0.006
Thickness 30 mm proximal to the insertion at the calcaneus bone in mm, mean (SD)	4.5 (0.8)	5.9 (0.8)	<0.001
Proportional relation between the thickness 30 mm proximal to the insertion at the calcaneus bone and maximal thickness in percent, mean	79	88	0.01
Structural alternations, edema, n (%)			0.40
Normal	28 (90.3)	9 (81.8)	
Light	3 (9.7)	1 (9.1)	
Moderate	-	1 (9.1)	
Severe	-	-	
Neovascularisation, n (%)			0.31
No	23 (74.2)	7 (63.6)	
Mild	7 (22.6)	3 (27.3)	
Moderate	1 (3.2)	1 (9.1)	
Severe			
Tendinosis, n (%)			0.47
No	22 (71.0)	7 (63.6)	
Yes	9 (29.0)	4 (36.4)	
Bursitis, n (%)			0.84
No	29 (93.5)	10 (90.9)	
Yes	2 (6.5)	1 (9.1)	
Qualitative morphology score	0.7 (1.2)	1.2 (1.4)	0.19

**Table 4:** Post hoc analyses of differences in thickness between asymptomatic (*n* = 31) and symptomatic Achilles tendons with the symptomatic tendons divided by observation of any morphological change (y/n), i.e., any structural alternation, neovascularization, sign of tendinosis, or bursitis. Morph. change = any morphological change observed at ultrasound examination.

Tendon Thickness	Asymptomatic Tendons *n* = 31	Symptomatic Tendons	
		Morph. Change	No Morph. Change
		*n* = 6	*n* = 5
Maximal (mm, mean (SD))	5.7 (1.0)	6.8 (1.1)	6.6 (0.4)
Diff. asymptomatic-symptomatic tendons	ref.	*p* = 0.04	*p* = 0.03
30 mm from insertion (mm, mean (SD))	4.5 (0.8)	6.1 (0.9)	5.6 (0.4)
Diff. asymptomatic-symptomatic tendons	ref.	*p* = 0.001	*p* = 0.02
Proportion 30 mm/Maximal (%, mean)	79	90	85
Diff. asymptomatic-symptomatic tendons	ref.	*p* = 0.01	*p* = 0.29
